# Immune Reactions Against Elongation Factor 2 Kinase: Specific Pathogenesis of Gastric Ulcer from *Helicobacter pylori* Infection

**DOI:** 10.1155/2009/850623

**Published:** 2009-07-14

**Authors:** Kiyoshi Ayada, Kenji Yokota, Yoshiro Kawahara, Yumiko Yamamoto, Kazuyuki Hirai, Tomoki Inaba, Masahide Kita, Hiroyuki Okada, Kazuhide Yamamoto, Keiji Oguma

**Affiliations:** ^1^Department of Bacteriology, Okayama University Graduate School of Medicine, Dentistry and Pharmaceutical Sciences, 2-5-1 Shikata-cho, Okayama 700-8558, Japan; ^2^Graduate School of Health Science, Okayama University, 2-5-1 Shikata-cho, Okayama 700-8558, Japan; ^3^Department of Medicine and Clinical Science, Okayama University Graduate School of Medicine, Dentistry and Pharmaceutical Sciences, 2-5-1 Shikata-cho, Okayama 700-8558, Japan; ^4^Department of Internal Medicine, Kagawa Prefectural Central Hospital, 5-4-16 Ban-cho, Takamatsu, Kagawa 760-8557, Japan

## Abstract

*Helicobacter pylori* (*H. pylori*) infection is a definite causative factor for gastric ulcers (GUs). In the present study we detected a specific antigen of gastric epithelial cells (HGC-27) using cell ELISA, which was recognized by the sera of GU patients (*n* = 20) but not in patients with chronic gastritis (CG; *n* = 20) or in healthy volunteers (HC; *n* = 10). This antigen was over-expressed by a stressful (heat-stressed) environment, and was identified as elongation factor 2 kinase (EF-2K) by western blotting. The GU patients' lymphocytes stimulated by *H. pylori* specifically disrupted heat-stressed HGC-27 cells in a cytotoxic assay. In flow cytometry, the effector cells (lymphocytes) from GU patients were significantly differentiated to T helper type 1 lymphocyte (Th1) and cytotoxic T lymphocyte (CTL) as opposed to those from CG patients. The target cells (HGC-27) expressed EF-2K and MHC-class I together with costimulatory molecules from heat stress. This antigen specific immune mechanism could have a prominent role in the pathogenesis of GU.

## 1. Introduction

*Helicobacter pylori *(*H. pylori*) infection causes acute gastritis [1, 2]. Persistent infection of *H. pylori *results in chronic gastritis with inflammatory infiltrates [3]. Multiple studies have demonstrated that *H. pylori* infection is associated with the pathogenesis of peptic ulcers, gastric adenocarcinoma, and mucosa-associated lymphoid tissue (MALT) lymphoma [4–6]. But not all patients infected by *H. pylori* experience these clinical manifestations. It has been proposed that the variety of pathologies depends on the virulent factors of *H. pylori*, such as Cag A, Vac A, Oip A [7–9]. Yet, the clinical consequences vary among patients infected by *H. pylori* possessing the same virulent genes [10]. Further, some studies have suggested that host factors, including the immune response to alkyl hydroperoxide reductase, and interleukin 1 (IL-1) gene family polymorphism, et al., might mainly affect the clinical outcome of *H. pylori* infection [11–13].

Our previous studies have demonstrated that *H. pylori* itself or its components elicited innate immune responses via toll-like receptor (TLR) 2 and TLR 4 on gastric epithelial cells and monocytes [14, 15]. Especially, IL-8 released from these cells is involved in the activation of neutrocytes and lymphocytes [13, 16]. These inflammatory participants release further cytokines such as IFN-*γ* and bring stress to the gastric epithelial cells. Indeed, overexpression of MHC class II dependent on IFN-*γ* [17] and heat shock proteins (HSPs) [18] were observed in gastric epithelia infected by *H. pylori*. Several recent studies have demonstrated that HSPs could help antigenic peptides (of self or foreign origin) transfer onto MHC molecules and that HSP-chaperoned antigenic peptides could induce antigen specific CTL and CD4^+^ T cells [19–21]. 

In our previous studies, we discovered the pathogenesis of mucosa associated lymphoid tissue (MALT) lymphoma evoked by autoimmune reactions against HSP60 [22, 23]. Further, we demonstrated that the heat stress mediated antigen could concern peptic ulcer. Peptic ulcers are a common disease caused by *H. pylori* infection and are easily improved by eradication in contrast to MALT lymphoma. Yet, the detailed mechanism of the pathogenesis of peptic ulcers is unclear. We herein identify the antigenic protein in gastric ulcer patients' sera and clarify the immune pathogenesis of peptic ulcers induced by this antigen.

## 2. Materials and Methods

### 2.1. Patients and Cells

Sera were obtained from patients suffering from gastric ulcers (GU; *n* = 20) and chronic gastritis (CG; *n* = 20) that consulted at the Okayama University Hospital and its associated hospitals. They received treatment with neither steroidal nor nonsteroidal anti-inflammatory drugs. All patients were diagnosed with CagA positive *H. pylori* infection by the detection of anti-CagA antibodies in their sera. Peripheral blood mononuclear cells (PBMCs) were also obtained from patients (GU; *n* = 8, CG; *n* = 8) before and after eradication therapy. The samples of healthy volunteers (HC; *n* = 10) who have not been infected by *H. pylori* were also obtained in the same way. This study was approved by the local ethics committee of each institute. Written informed consent was obtained from each patient. Diagnosis of these diseases was based on the findings of a gastroduodenal endoscopy and histology of biopsy specimens.

We employed the human gastric cell line HGC-27, which was established from a gastric cancer patient (kindly provided by Professor Tadashi Yoshino, Okayama University, Japan).

### 2.2. Cell ELISA and Western Blotting for Autoantibodies

Serum autoantibody levels against gastric cells were measured by cell ELISA as in a previous study [23]. In brief, HGC-27 cells were cultured in 96-well microtiter plates as a monolayer. Adherent cells in plates were washed with PBS and fixed by 2% formaldehyde in PBS. The cells in an additional plate were induced to express stress-oriented proteins by heat treatment at 42°C for 10 minutes. After heat treatment, the cell viabilities were checked by microscopy. Since the induction of stress proteins in HGC-27 cells was at a maximum 1 hour after heat stress, HGC-27 cells were incubated and then fixed as previously mentioned [23]. The two plates of cells (nontreated and heat treated) were incubated with PBS containing 10% skim milk in order to prevent nonspecific reactions. The patients' sera, which were diluted with PBS containing 10% skim milk, reacted with both plates of cells for 2 hours at room temperature and then washed with PBS three times. Autoantibodies against gastric cells were detected by a peroxidase conjugated antihuman IgG and a substrate of *o*-phenylenediamine (OPD, Dako Pure Chemical Industries Ltd., Osaka, Japan) in a citrate buffer (pH 5.5). The color developments in the plates were measured using a BioRad plate reader. 

The antigens of autoantibodies were analyzed by Western blotting. HGC-27 cells were treated at 42°C for 10 minutes and then were incubated at 37°C for 1 hour. Nonheat-treated cells were employed as a control. The proteins of the HGC-27 cells were extracted with a Subcellular Proteome Extraction Kit (Merck Ltd. Japan, Tokyo, Japan) in accordance with the manufacturer's protocol. Fractions of cell membranes and cytoplasm were employed. Both (heat-stressed and nonheat-stressed) cell fractions were subjected to SDS-PAGE using 10% running gels. The proteins were then transferred to PVDF membranes (Nihon Millipore K.K., Tokyo, Japan). The membranes were washed three times with PBS for five minutes and blocked with PBS containing 10% skim milk for 1 hour at room temperature. After washing with PBS containing Tween 20 (0.1%, v/v) (PBST), the membranes were incubated with human sera diluted to 1:100 in PBS containing 10% skim milk overnight at 4°C. Following three washes with PBST, the blots were incubated with an antihuman IgG HRP-linked antibody (Dako, Kyoto, Japan) for 2 hours at room temperature. After three washes with PBST, bands were visualized with ECL Western blotting detection reagents (GE Healthcare UK Ltd., Buckinghamshire, England) and exposure to an LAS-1000 mini Bio-imaging Analyzer System (FUJIFILM Co., Tokyo, Japan). The band recognized only by the sera of GU patients was separated. The amino acid sequences were then analyzed with an Applied Biosystems protein sequencer model 491HT (Applied Biosystems Inc., Foster City, Calif, USA). HGC27 cells infected by *H. pylori *ATCC43504 strain were also used in Western blotting, as previously mentioned [15]. EF-2K was detected by antihuman EF-2K antibody (Cell Signaling Technology, Danvers, Mass, USA).

### 2.3. Cloning, Expression, and Purification of Recombinant Protein, EF-2K and ELISA for Antibodies Against EF-2K

The putative antigen of autoantibody, elongation factor 2 kinase (EF-2K), was expressed as recombinant protein. Total RNA was extracted from HGC-27 cells (3 × 10^6^ cells) using an RNeasy Mini kit (Qiagen K.K., Tokyo, Japan). The concentration and purity of the RNA were determined by measuring the A_260_ and the A_260_/A_280_ ratio with a Beckman DU-7000 (Beckman Coulter, Inc., Fullerton, Calif, USA). Total cellular RNA (1 *μ*g/5 *μ*L) was reverse transcribed using a superscript III transcription kit (Invitrogen Co., Carlsbad, Calif, USA). The EF-2K gene was amplified by primer (forward: ATC ACC ATC ACC ACG GTA TGG CAG ACG AAG ATC TCA TC, reverse: CTT GGT TAG TTA GTT ATT ACT CCT CCA TCT GGG CC) designed to annex a histidine tag to the N-terminal, using an EasyXpress Linear Template kit (Qiagen K.K., Tokyo, Japan). Protein was expressed by an EasyXpress Protein Synthesis kit (Qiagen K.K., Tokyo, Japan). Soluble fusion proteins were purified with a nickel column (GE Healthcare UK Ltd., Buckinghamshire, England) by affinity chromatography. All procedures were performed according to the manufacturers' instructions. DNA and protein sequences were verified in each step. 

Serum titers of IgG antibodies against EF-2K were measured by ELISA. 96-well microtiter plates were coated with recombinant EF-2K proteins (10 *μ*g/mL) in 100 *μ*L of 0.1 M carbonate-bicarbonate buffer (pH 9.6) onto the wells of the microtiter plate overnight at 4°C. The wells were first washed with PBST. Then the wells were blocked with 200 *μ*L of PBS containing 10% skim milk (dilution buffer) for 2 hours at room temperature. After washing with PBST, the plates were incubated with 100 *μ*L of patients' sera at 1:1000 dilution for 2 hours at room temperature. After being washed with PBST, 100 *μ*L of peroxidase labeled rabbit antihuman IgG antibody (DAKO, Kyoto, Japan) at 1:3000 dilution or peroxidase labeled mouse antihuman IgG1 or IgG2 antibodies (ZYMED, Carlsbad, CA) at 1:1000 dilution was added, followed by incubation for 2 hours at room temperature. After the plates were washed, the wells reacted with 150 *μ*L of 1 mg of OPD per mL in citrate buffer (pH 5.5). Then 50 *μ*L of 2N H_2_SO_4_ was added to stop the reaction and the OD was measured at 490 nm.

### 2.4. Cytotoxicity Assay

Cytotoxicity assays of patients' lymphocytes (effector cells) against HGC-27 cells (target cells) were examined.

Peripheral blood was obtained from the GU patients (*n* = 8) before and after the eradication of *H. pylori*, and also from the CG patients (*n* = 8). Mononuclear cells (PBMCs) were isolated from the blood using a Ficol-Paque (GE Healthcare UK Ltd., Buckinghamshire, England) and then divided into four aliquots. 1 × 10^6^ PBMCs were seeded in 24-well microtiter plates with an RPMI-1640 medium supplemented with 10% FCS in a humidified 37°C, 5% CO_2_ incubator for 14 days. The first aliquot was cultured with sonic extracted *H. pylori* ATCC43504 antigens (*H. pylori* lysate) (5 *μ*g/mL) and concanavalin A (con A) (2 *μ*g/mL) (SIGMA-ALDRICH Japan K.K., Tokyo, Japan). The second was cultured with *H. pylori* lysate, con A, and IL-12 (0.2 ng/mL) (R&D systems, Minneapolis, Minn, USA). The third was cultured with *H.pylori* lysate, con A, and IL-4 (0.2 ng/mL) (R&D systems, Minneapolis, Minn, USA). The forth was used as a control. IL-12 was used for the induction of Th-1 dominant immunity, and IL-4 was used for Th-2 immunity. The lymphocytes were selected by the nylon wool method and then used as effector cells.

HGC-27 cells were maintained in a DMEM medium supplemented with 10% FCS in a humidified 37°C, 5% CO_2_ incubator. The cells were divided into four aliquots. The first aliquot was subjected to heat stress at 42°C for 10 minutes, the second reacted with sera from the patients for 1 hour, the third was given both treatments, and the fourth served as a control. The sera were treated at 56°C for 30 minutes to inactivate complement before cell treatment. Cells were washed twice, suspended with a RPMI-1640 medium supplemented with 10% FCS, and then used as target cells.

Effector cells and target cells reacted with various effector/target (E/T) ratios for 4 hours, and then cytotoxicities were evaluated with Cytotox 96 (Promega K.K., Tokyo, Japan) according to the levels of LDH released from disrupted target cells. The percentage of cytotoxicity was calculated as follows: [(experimental value − effector spontaneous value − target spontaneous value)/(target maximum value − target spontaneous value)] × 100.

### 2.5. Flow Cytometry Analysis

The populations of T lymphocyte subsets were analyzed with flow cytometry. Cell surface antigens (CD4 or CD8) and cytoplasmic cytokines (IFN-*γ* and IL-4) were stained with fluorescent labeled antibodies following the manufacturer's instructions. In brief, the cells were stimulated with phorbol 12-myristate 13-acetate (PMA) (50 ng/mL) and ionomycin (500 ng/mL) (SIGMA-ALDRICH Japan K.K., Tokyo, Japan) for 6 hours and reacted with brefeldin A (10 *μ*g/mL) (Becton, Dickinson and Company, Franklin Lakes, NJ, USA) for 2 hours before harvest. Cell surface antigens were stained with PE-Cy5 conjugated mouse antihuman CD4 or CD8 antibodies (Becton, Dickinson and Company, Franklin Lakes, NJ, USA). After permeabilization and fixation, cells were stained with FITC conjugated mouse antihuman IFN-*γ* and PE conjugated mouse antihuman IL-4 antibodies (Becton, Dickinson and Company, Franklin Lakes, NJ, USA). 

The expression of antigens in target cells which were recognized in ulcer patient's sera was confirmed by flow cytometry. Heat-stressed cells reacted with ulcer patients' sera for 1 hour on ice and then reacted with FITC conjugated mouse antihuman IgG antibody. Further, the expression of HLA-A, B, C and costimulatory molecules (CD80, CD86) were detected by PE-Cy5 conjugated mouse antihuman HLA-A, B, C antibody, FITC conjugated mouse antihuman CD80 antibody, and PE conjugated mouse antihuman CD86 antibody (Becton, Dickinson and Company, Franklin Lakes, NJ, USA). After fixation with 2% formaldehyde PBS, cells were analyzed by a BD FACScan flow cytometry system (Becton, Dickinson and Company, Franklin Lakes, NJ, USA).

### 2.6. Statistical Analysis

All statistical analyses were performed using Kaleida Graph software (Synergy Software, Reading, Pa, USA). The difference among the groups was analyzed by the Wilcoxon-Mann-Whitney test. *P* < .05 was considered to be significant.

## 3. Results

### 3.1. Autoantibodies Against Gastric Cells in Patients

**Figure 1:**
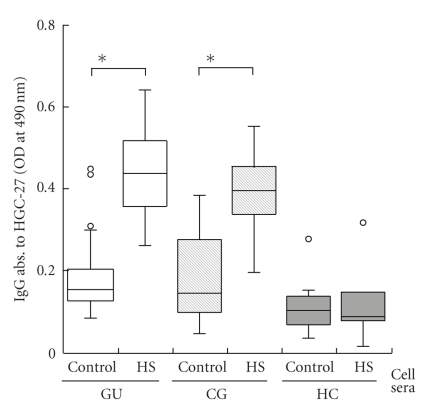
*Autoantibody to heat-stressed HGC-27 cells in GU patients*. (a) The titers of anti-HGC27 antibodies in patients with *H. pylori* infection and healthy volunteers are shown. The open column indicates the antibody titer in GU patients, the striped is CG patients, and the solid is HC. The left column displays the titers of anti-nonheat-stressed HGC27 antibody and the right antiheat-stressed HGC27 antibody in each group. The titers of anti-HGC-27 antibody were significantly elevated by heat stress in GU and CG patients (*P < .01).

Autoantibodies to gastric epithelial cells were detected by cell-ELISA in GU and CG patients infected by *H. pylori* and the HC group. The titers of antibodies against HGC-27 cells in GU and CG patients significantly increased when using heat-stressed cells as antigens compared to nonheat-stressed cells, while those in the HC group did not (Figure 1).

This result followed our previous study [23] and suggested that *H. pylori* infection elicited immune reactions against some host's antigens which were induced by heat stress.

### 3.2. Heat Stress-Induced Antigens in Gastric Epithelial Cells

**Figure 2:**
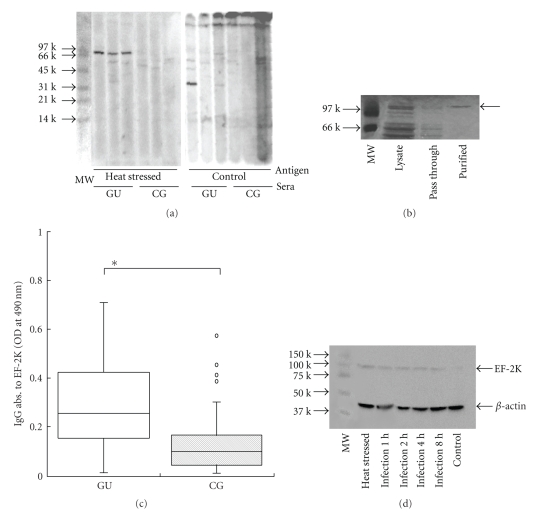
*The putative antigen in heat-stressed HGC-27 cells was detected as EF-2K*. (a) Western blotting of HGC-27 cells and heat-stressed HGC-27 cells by the sera of GU and CG. Lane 1; molecular weight marker, lanes 2–4; the proteins from heat-stressed HGC-27 cells reacted by GU sera, lanes 5–7; the proteins from heat-stressed HGC-27 cells reacted by CG sera, lanes 8–10; the proteins from nonstressed HGC-27 cells reacted by GU sera, lanes 11–13; the proteins from nonstressed HGC-27 cells reacted by CG sera. 90 kDa protein from heat-stressed HGC-27 cells reacted by GU sera specifically. (b) The expression and purification of EF-2K recombinant protein. About 100 kDa protein was purified. (c) Antibody to EF-2K recombinant protein in patients with GU and CG. The titer of anti-EF-2K recombinant protein antibodies in GU was significantly higher than that in CG (*P < .05). (d) Quantification of EF-2K expressions in HGC-27 infected by *H. pylori*. The EF-2K expressions in HGC-27 were enhanced by *H. pylori* infection as by heat stress.

The antigens induced by heat stress in gastric epithelial cells were analyzed by Western blotting. The 90 kDa antigen extracted from heat-stressed HGC-27 was recognized by all of the GU patients' sera but not by the CG group at all (Figure 2(a)). Nonheat-stressed antigens (control) did not express common antigens recognized by any patients' sera. Therefore, only GU patients had immune reactivity to this 90 kDa protein which was generated in HGC-27 with a stressful state. Amino acids sequence analysis revealed that the N-terminal amino acid sequence of this 90 kDa antigen was MADEDLIFRL, which was identical to that of elongation factor 2 kinase (EF-2K) (NP_037434). The recombinant protein expressed and purified as EF-2K was also verified as 100 kDa protein in SDS-PAGE (Figure 2(b)). The titer of anti-EF-2K recombinant protein antibody of GU patients was significantly higher than that of CG patients (Figure 2(c)). The subclass of IgG in the anti-EF-2K recombinant protein antibodies of GU patients was IgG2 (data not shown). These results suggested that the immune reaction against EF-2K was causative of GU. To evaluate the EF-2K expressions in HGC-27 cells infected by *H. pylori*, the amounts of EF-2K in HGC-27 cells cocultured with *H. pylori* were quantified. *H. pylori* infection enhanced the EF-2K expressions in HGC-27 cells as much as heat stress did, regardless of the duration of the infection (Figure 2(d)). This result suggested that the effect of heat stress to the gastric epithelia mimicked that of infection by *H. pylori*.

### 3.3. Cytotoxicity Against Gastric Cells by Patients' Lymphocytes

**Figure 3:**
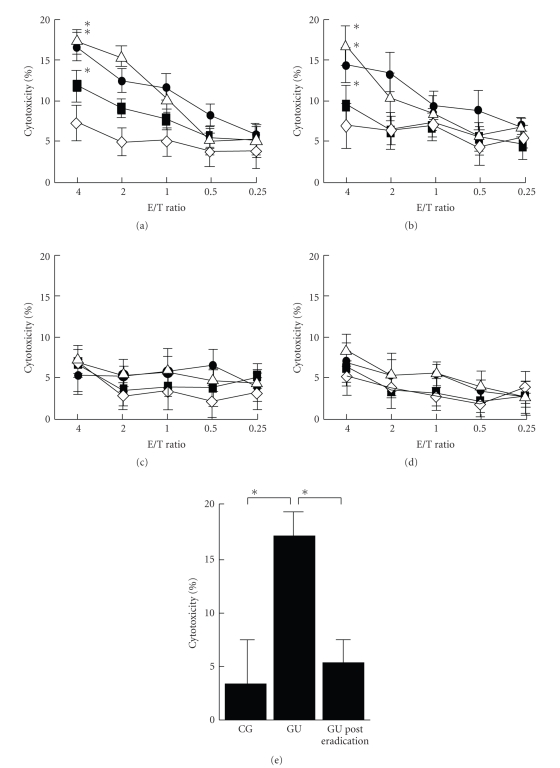
*Specific cytotoxicity induced by lymphocytes from GU patients.* 4 target cells were prepared. (a) HGC-27 cells treated with heat stress and patients' sera, (b) HGC-27 cells treated with heat stress, (c) HGC-27 cells treated with patients' sera, (d) nontreated HGC-27 cells. PBMCs were taken from GU patients (*n* = 8). PBMCs stimulated with conA and *H. pylori* (■) in addition to IL-12 (∆) or IL-4 (●) were used as effector cells. Nonstimulated PBMCs (◊) were also used as a control. Both cells reacted with various E/T ratios. An E/T ratio dependent cytotoxicity was observed only against heat-stressed HGC-27 cells or heat-stressed and sera-treated HGC-27 cells. The cytotoxicities of effector cells stimulated by *H. pylori* lysate were significantly enhanced, regardless of additional cytokine stimulations (*; *P* < .05). (e) The comparison of cytotoxicities among lymphocytes from CG, GU, and GU posteradication patients. Target cells were HGC-27 cells treated with heat stress and patients' sera. Effector cells were PBMCs stimulated with conA, *H. pylori*, and IL-12. The E/T ratio was 4. Specific cytotoxicity was only observed in GU patients' lymphocytes.

We evaluated whether lymphocytes from the GU patients possessed any cytotoxicity against gastric cells. Specific cytotoxicity of the lymphocytes, which was dependent on the E/T ratio, was observed only when the heat-stressed HGC-27 cells or heat-stressed and sera-treated HGC-27 cells were employed as target cells (Figures 3(a), 3(b), 3(c), and 3(d)). Further, the patients' sera did not affect the cytotoxicities. These results suggested that this cytotoxity could target the antigenic peptide of EF-2K which was overexpressed on HGC-27 cells by heat stress and that the antibody-dependent cell-mediated cytotoxicity (ADCC) did not occur. Effector cells stimulated by *H. pylori* lysate and additional cytokines had significantly higher cytotoxicities than the control effecter cells (Figures 3(a) and 3(b)).

The effector cells obtained from CG did not have cytotoxicity. Further, lymphocytes taken from GU patients 6 months after successful eradication therapy lost the cytotoxicity (Figure 3(e)).

### 3.4. FACScan Analysis of Effector Cells and Target Cells

**Figure 4:**
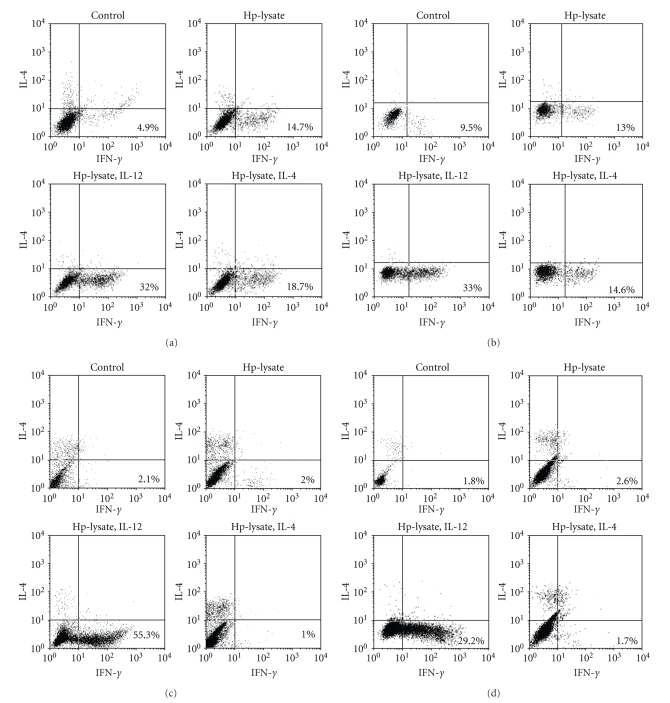
*Flow cytometric analyses of effector cells*. Typical subsets analysis of CD4^+^ (a) and CD8^+^ (b) lymphocytes from GU patients, and CD4^+^ (c) and CD8^+^ (d) lymphocytes from CG patients are shown. *H. pylori* lysate induced the expression of IFN-*γ* in CD4^+^ cells regardless of additional cytokine stimulations, while CG indicated the expression of IFN-*γ* in CD4^+^ cells only by IL-12 stimulation. The same tendency was observed in CD8^+^ cells.

To determine the subsets of the lymphocytes, effector cells were employed in a FACScan analysis. Typical analysis data from patients with GU indicated that stimulation with *H. pylori* lysate induced the expression of IFN-*γ* in CD4^+^ cells regardless of additional cytokine stimulations (Figure 4(a)), while data from CG patients indicated the expression of IFN-*γ* in CD4^+^ cells only by additional IL-12 stimulation (Figure 4(c)). Next, we analyzed the subsets of CD8^+^ cells (Figures 4(b) and 4(d)). In CD8^+^ cells, the same tendency as CD4^+^ cells was observed. The CD8^+^ cells were significantly induced by *H. pylori* lysate stimulation regardless of additional cytokine stimulations in the GU patients.

**Figure 5:**
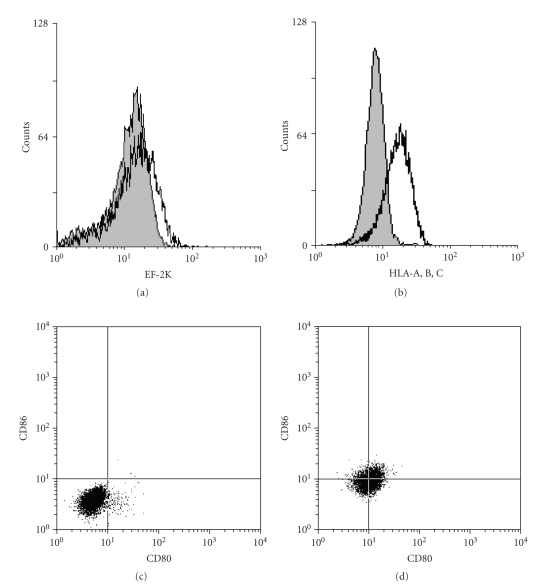
*Flow cytometric analyses of target cells*. (a) Typical analysis of EF-2K expression on HGC-27 cells is shown. The shadowed histogram indicates the EF-2K detected by GU sera in nontreated HGC-27 cells. And the bold histogram indicates the EF-2K detected by GU sera in heat-stressed HGC-27 cells. (b) Typical analysis of MHC class I molecule expression on HGC-27 cells is shown. The shadowed histogram indicates MHC class I molecules detected by PE-Cy5 conjugated mouse antihuman HLA-A, B, C antibody in nontreated HGC-27 cells and the bold histogram indicates MHC class I molecules detected by PE-Cy5 conjugated mouse antihuman HLA-A, B, C antibody in heat-stressed HGC-27 cells. The costimulatory molecules detected by FITC conjugated mouse antihuman CD80 antibody and PE conjugated mouse antihuman CD86 antibody on nontreated HGC-27 cells are shown in (c) and those of heat-stressed HGC-27 cells in (d). The heat stress enhanced the expression of EF-2K, MHC class I molecules, and costimulatory molecules on HGC-27 cells (target cells).

In the target cells, EF-2K was detected with GU patients' sera only in cases of heat stress treatment (Figure 5(a)). Further, the heat stress also enhanced the expression of MHC class I molecules and costimulatory molecules in target cells (Figures 5(b), 5(c), and 5(d)).

## 4. Discussion

The pathogenesis of peptic ulcers is complicated because of the involvement of multiple factors; however, the association with *H. pylori* is definite [4]. Although bacterial virulent factors have been identified and their pathogenic mechanisms have been clarified [7–9], the specific host's mechanism of gastric ulcers is still obscure. Innate immune responses toward *H. pylori* sustained the stress against gastric epithelial cells [14]. The stress-mediated protein might be the target in the pathogenesis of gastric ulcers as HSP60 was in MALT lymphoma [22–24]. This study aimed to shed light on the target protein affected by stress and the mechanism by which adaptive immune responses induced gastric ulcers specifically.

In the present study, we demonstrated that the sera of patients infected by *H. pylori* reacted to heat-stressed epithelial cells, while those of the healthy control group did not (Figure 1). This result suggested that *H. pylori* infection could provoke the host to generate the autoimmunity against some self proteins which were overexpressed by heat stress in the gastric epithelium. Further investigations revealed that gastric ulcer patients have antibodies which recognize the protein expressed in stressed epithelial cells. We report here that elongation factor 2 kinase (EF-2K) could be a candidate for the epitopic protein of antibodies in gastric ulcer patients (Figure 2).

Intrinsically, EF-2K phosphorylates and inactivates elongation factor 2 (EF-2), resulting in the inhibition of peptide-chain elongation [25]. EF-2K is chaperoned by heat shock protein 90 (HSP90) and its activities are regulated by Ca2^+^ ions and calmodulin [26, 27]. Also, EF-2K is affected by cAMP-dependent protein kinase in response to elevated cAMP levels, which were increased in the stressed condition [28]. HSP90 also contributes to not only the transduction of steroidal hormone signals but also to the transport of endogenous antigens in cytoplasm [29, 30]. In the present study, heat stress might affect EF-2K abnormally and induce the expression of antigenic peptide derived from EF-2K in HGC-27 cells. Since HSP90 was not detected on the HGC-27 cell surface (data not shown), the whole molecule of EF-2K complexed with HSP90 was not presented. More detail about the antigen presenting procedure is unknown and should be clarified in following studies.

In prokaryote including *H. pylori*, elongation factor G (EF-G) is the counterpart of EF-2 in eukaryotic cells. And EF-G is also phosphorylated and inactivated by ribosomal enzymes, for example, by PrkC of *Bacillus subtlis* [31]. Since the threonines in these two factors were phosphorylated by each kinase equally [25, 31], the amino acid sequences of EF-2K could be highly identical to those of EF-G kinase in the enzymatic active site. Therefore, this molecular mimicry might elicit the “autoimmunity” which generated antibodies against self EF-2K.

In order to evaluate the pathogenesity of anti-EF-2K antibodies, the cytotoxic activities were investigated. GU patient's sera themselves did not have antibody-complement mediated cytotoxicities against heat-stressed gastric cells. In contrast, the cytotoxic activities of GU patients' lymphocytes were observed only against heat-stressed HGC-27 cells regardless of treatment with patients' sera (Figures 3(a)–3(d)). These results suggest that anti-EF-2K antibodies do not have a direct effect on gastric epithelial cells, but that lymphocytes, which recognize antigenic peptide expressed by heat stress (i.e., EF-2K peptide), play a dominant role in cytotoxic effects. These cellular-dominant immune responses were confirmed by the detection of a subclass of anti-EF-2K IgG2 antibodies.

The effector cells of GU patients, which were stimulated with *H. pylori* antigens, enhanced their cytotoxicities more strongly than those with no stimulation. The additional stimulation of effector cells with IL-12 or IL-4 brought similar enhancement compared to stimulation with only *H. pylori* antigens (Figure 3(e)). The Th1/Th2 balance, which was inclined to Th1, did not differ between GU and CG in IL-12 stimulated effector cells, and the population of CD8^+^ and IFN-*γ* positive lymphocytes significantly increased in GU patients' effector cells stimulated with *H. pylori* antigens. These parallel relations between cytotoxicities and the population of CD8^+^ imply that *H. pylori* antigens specific cytotoxic CD8^+^ T lymphocytes cross-reacted against heat-stressed epithelial cells and disrupted them. Further, CD8^+^ T lymphocytes' cross-reactivities were observed only in GU patients' lymphocytes (Figure 3(e)). These enhanced cytotoxicities against gastric epithelial cells would result in a gastric ulcer in vivo.

In this study, we also revealed the differences in cytotoxicities between the lymphocytes from gastric ulcer patients before and after eradication therapy. The lymphocytes after eradication therapy diminished their cytotoxicity significantly (Figure 3(e)). This result indicates that the resting memory of CD8^+^ cells is not fully activated by only 2 weeks of *H. pylori* antigen stimulation.

In conclusion, we identified a cytotoxic immune response in gastric ulcer patients infected by *H. pylori*. In this immune response, *H. pylori* antigens specific CD8^+^ T lymphocytes could damage gastric epithelial cells which overexpressed EF-2K. These autoreactive lymphocytes are generated only in ulcer patients with *H. pylori *infection. Our investigation will contribute to the realization of the pathogenesis of gastric ulcers caused by *H. pylori* infection.
